# Crisis Intervention Team of Avilés. Results after three years

**DOI:** 10.1192/j.eurpsy.2022.1555

**Published:** 2022-09-01

**Authors:** A.M. González Álvarez, E. Lago Machado, L. Pérez Gómez, E. Lanza Quintana, J.J. Martínez Jambrina

**Affiliations:** 1 Servicio de Salud del Principado de Asturias, Centro De Salud Mental La Magdalena, Avilés, Spain; 2 Servicio de Salud del Principado de Asturias, Hospital San Agustin, Avilés, Spain

**Keywords:** crisis intervention team Aviles

## Abstract

**Introduction:**

This Crisis Intervention Team was born in October 2018 with the aim of intensifying the treatment of people in psychiatric crisis situation.

**Objectives:**

Provide an intensive and early assessment and approach in a timely manner. It also provides home care if necessary.

**Methods:**

The team intensively performs scheduled visits, emergencys, telephone interventions and home care. It is in constant coordination with other structures of the mental health and socio-health network.

**Results:**

A total of 83 patients have been included in our team since its inception. The youngest was 17 years old and the oldest 83 years old (exceptional case in evaluation). The mean age was 45.6 years. 67.4% were female (56 women) and 32.5% male (27 men). The delay in care did not exceed 48 hours.

200 patients were evaluated into suicide protocol, with ages ranging from 15 to 85 years, with a mean age of 45.4 years. The delay in care does not exceed 10 days.

**Conclusions:**

This is a team that offers a rapid response, dedicates the necessary time for a correct evaluation of the risk, of the evolution and tries to establish a therapeutic alliance in record time. It is able to tolerate a certain degree of uncertainty, manage and tolerate the level of risk. He stands out for being flexible and dynamic in order to be able to adapt to the patients and theirs circumstances. This requires empathy, closeness and commitment.

**Disclosure:**

No significant relationships.

